# Supplementary material for: Causal effect of atrial fibrillation/flutter on chronic kidney disease: A bidirectional two-sample Mendelian randomization study
Source: PLoS One. 2021 Dec 13;16(12):e0261020. doi: 10.1371/journal.pone.0261020 (PMC8668124; doi:10.1371/journal.pone.0261020)
Supplement: S1 Checklist — (DOC) [file pone.0261020.s001.doc]

# STREGA reporting recommendations, extended from STROBE Statement

| **Item** | **Item number** | **STROBE Guideline** | **Extension for Genetic Association Studies (STREGA)** | **Page number** |
| --- | --- | --- | --- | --- |
| **Title and Abstract** | 1 | (a) Indicate the study’s design with a commonly used term in the title or the abstract. |  | Pages 1 to 2 |
|  |  | (b) Provide in the abstract an informative and balanced summary of what was done and what was found. |  | Pages 1 to 2 |
| **Introduction** | | |  |  |
| *Background rationale* | 2 | Explain the scientific background and rationale for the investigation being reported. |  | Pages 3 to 4 |
| *Objectives* | 3 | State specific objectives, including any pre-specified hypotheses. | ***State if the study is the first report of a genetic association, a replication effort, or both.*** | Page 4 |
| **Methods** | | |  |  |
| *Study design* | 4 | Present key elements of study design early in the paper. |  | Page 4 |
| *Setting* | 5 | Describe the setting, locations and relevant dates, including periods of recruitment, exposure, follow-up, and data collection. |  | Pages 5 to 6 |
| *Participants* | 6 | 1. **Cohort study –** Give the eligibility criteria, and the sources and methods of selection of participants. Describe methods of follow-up.   **Case-control study –** Give the eligibility criteria, and the sources and methods of case ascertainment and control selection. Give the rationale for the choice of cases and controls.  **Cross-sectional study –** Give the eligibility criteria, and the sources and methods of selection of participants. | ***Give information on the criteria and methods for selection of subsets of participants from a larger study, when relevant***. | Pages 5 to 6 |
|  |  | 1. **Cohort study –** For matched studies, give matching criteria and number of exposed and unexposed.   **Case-control study –** For matched studies, give matching criteria and the number of controls per case. |  |  |
| *Variables* | 7 | *(a)* Clearly define all outcomes, exposures, predictors, potential confounders, and effect modifiers. Give diagnostic criteria, if applicable. | ***(b)*** ***Clearly define genetic exposures (genetic variants) using a widely-used nomenclature system. Identify variables likely to be associated with population stratification (confounding by ethnic origin).*** | Pages 4 to 6 |
| *Data sources measurement* | 8***** | *(a)* For each variable of interest, give sources of data and details of methods of assessment (measurement). Describe comparability of assessment methods if there is more than one group. | ***(b)*** ***Describe laboratory methods, including source and storage of DNA, genotyping methods and platforms (including the allele calling algorithm used, and its version), error rates and call rates. State the laboratory/centre where genotyping was done****.* ***Describe comparability of laboratory methods if there is more than one group. Specify whether genotypes were assigned using all of the data from the study simultaneously or in smaller batches.*** | Not available as all dataset we used were publicly available from CKDGen and UK Biobank.  We did not analyse any DNA in laboratory. |
| *Bias* | 9 | *(a)* Describe any efforts to address potential sources of bias. | ***(b) For quantitative outcome variables, specify if any investigation of potential bias resulting from pharmacotherapy was undertaken. If relevant, describe the nature and magnitude of the potential bias, and explain what approach was used to deal with this.*** | Pages 5 to 9 |
| *Study size* | 10 | Explain how the study size was arrived at. |  | Pages 5 to 6 |
| *Quantitative variables* | 11 | Explain how quantitative variables were handled in the analyses. If applicable, describe which groupings were chosen, and why. | ***If applicable, describe how effects of treatment were dealt with.*** | Pages 6 to 9 |
| Statistical methods | 12 | (a) Describe all statistical methods, including those used to control for confounding. | ***State software version used and options (or settings) chosen.*** | Page 4  Pages 6 to 9 |
|  |  | (b) Describe any methods used to examine subgroups and interactions. |  | Not available |
|  |  | (c) Explain how missing data were addressed. |  | Not available |
|  |  | 1. **Cohort study –** If applicable, explain how loss to follow-up was addressed.   **Case-control study –** If applicable, explain how matching of cases and controls was addressed.  **Cross-sectional study –** If applicable, describe analytical methods taking account of sampling strategy. |  |  |
|  |  | (e) Describe any sensitivity analyses. |  | Pages 8 to 9 |
|  |  |  | ***(f) State whether Hardy-Weinberg equilibrium was considered and, if so, how****.* | No, as we used only publicly available GWAS summary data. |
|  |  |  | ***(g) Describe any methods used for inferring genotypes or haplotypes.*** | We did not infer genotypes or haplotypes. |
|  |  |  | ***(h) Describe any methods used to assess or address population stratification.*** | Pages 5 to 6  Only European ancestry |
|  |  |  | ***(i) Describe any methods used to address multiple comparisons or to control risk of false positive findings.*** | Pages 8 to 19  Sensitivity analyses |
|  |  |  | ***(j) Describe any methods used to address and correct for relatedness among subjects*** | Pages 5 to 6  Avoid participant overlap |
| **Results** | | |  |  |
| *Participants* | 13***** | 1. Report the numbers of individuals at each stage of the study – e.g., numbers potentially eligible, examined for eligibility, confirmed eligible, included in the study, completing follow-up, and analysed. | ***Report numbers of individuals in whom genotyping was attempted and numbers of individuals in whom genotyping was successful.*** | Not available as all dataset we used were publicly available from CKDGen and UK Biobank. |
|  |  | (b) Give reasons for non-participation at each stage. |  | Not available |
|  |  | (c) Consider use of a flow diagram. |  | Not available |
| *Descriptive data* | 14***** | (a) Give characteristics of study participants (e.g., demographic, clinical, social) and information on exposures and potential confounders. | ***Consider giving information by genotype****.* | We did not analyse genotype. |
|  |  | (b) Indicate the number of participants with missing data for each variable of interest. |  | Not available |
|  |  | 1. **Cohort study –** Summarize follow-up time, e.g. average and total amount. |  |  |
| *Outcome data* | 15 ***** | **Cohort study-**Report numbers of outcome events or summary measures over time. | ***Report outcomes (phenotypes) for each genotype category over time*** | We did not analyse genotype. |
|  |  | **Case-control study –** Report numbers in each exposure category, or summary measures of exposure. | ***Report numbers in each genotype category*** | We did not analyse genotype. |
|  |  | **Cross-sectional study –** Report numbers of outcome events or summary measures. | ***Report outcomes (phenotypes) for each genotype category*** | We did not analyse genotype. |
| *Main results* | 16 | (a) Give unadjusted estimates and, if applicable, confounder-adjusted estimates and their precision (e.g., 95% confidence intervals). Make clear which confounders were adjusted for and why they were included. |  | Pages 9 to 15  Confounders checked by PhenoScanner were excluded |
|  |  | (b) Report category boundaries when continuous variables were categorized. |  | Not available |
|  |  | (c) If relevant, consider translating estimates of relative risk into absolute risk for a meaningful time period. |  | Not available |
|  |  |  | ***(d) Report results of any adjustments for multiple comparisons.*** | Pages 9 to 15 |
| *Other analyses* | 17 | 1. Report other analyses done – e.g., analyses of subgroups and interactions, and sensitivity analyses. |  | Pages 9 to 15 |
|  |  |  | ***(b) If numerous genetic exposures (genetic variants) were examined, summarize results from all analyses undertaken.*** | Page 16  In the beginning of Discussion section. |
|  |  |  | ***(c) If detailed results are available elsewhere, state how they can be accessed.*** | Not available |
| **Discussion** | | |  |  |
| *Key results* | 18 | Summarize key results with reference to study objectives. |  | Page 16 |
| *Limitations* | 19 | Discuss limitations of the study, taking into account sources of potential bias or imprecision. Discuss both direction and magnitude of any potential bias. |  | Pages 17 to 18 |
| *Interpretation* | 20 | Give a cautious overall interpretation of results considering objectives, limitations, multiplicity of analyses, results from similar studies, and other relevant evidence. |  | Pages 16 to 18 |
| *Generalizability* | 21 | Discuss the generalizability (external validity) of the study results. |  | Page 18 |
| **Other Information** | | |  |  |
| *Funding* | 22 | Give the source of funding and the role of the funders for the present study and, if applicable, for the original study on which the present article is based. |  | We have no funding source. |

STREGA = STrengthening the REporting of Genetic Association studies; STROBE = STtrengthening the Reporting of Observational Studies in Epidemiology.

* Give information separately for cases and controls in case-control studies and, if applicable, for exposed and unexposed groups in cohort and cross-sectional studies.
