# Supplementary figures and images for: Causal effect of atrial fibrillation/flutter on chronic kidney disease: A bidirectional two-sample Mendelian randomization study
Source: PLoS One. 2021 Dec 13;16(12):e0261020. doi: 10.1371/journal.pone.0261020 (PMC8668124; doi:10.1371/journal.pone.0261020)

## Slide 1
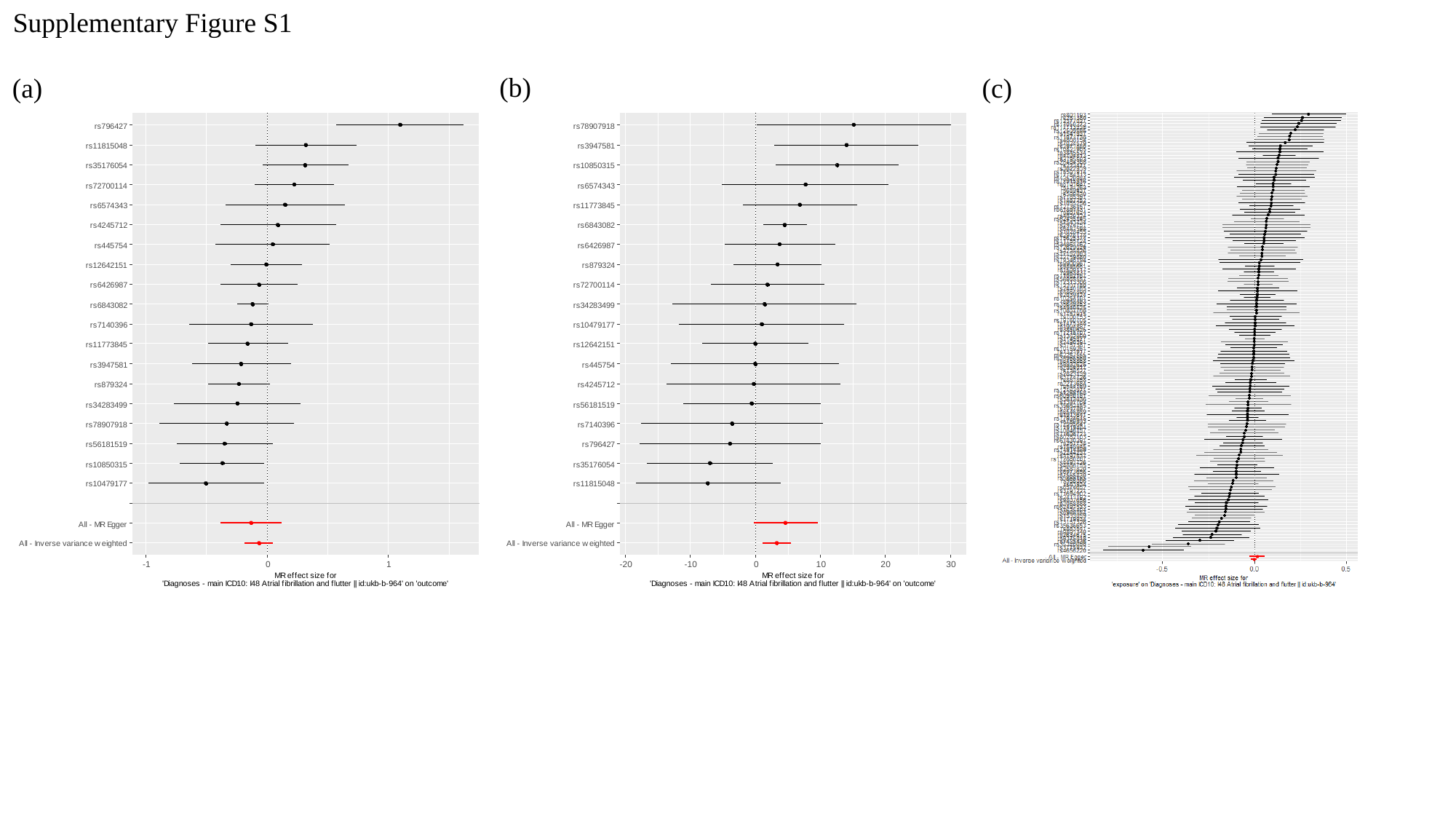

Supplementary Figure S1
(b)
(c)
(a)

Supplement: S1 Fig — (a) Forrest plot for estimating the risk of AF/F on the change in eGFR. (b) Forrest plot for estimating the causal effect of the risk of AF/F on the risk of CKD. (c) Forrest plot for estimating the causal effect of the change in eGFR on the risk of AF/F. Each black point represents the causal estimate of each SNP on the outcome per increase in the exposure, and red points show the combined causal estimates using IVW and MR-Egger regression methods with horizontal lines denoting 95% confidence intervals. (PPTX) [file pone.0261020.s002.pptx]

## Slide 1
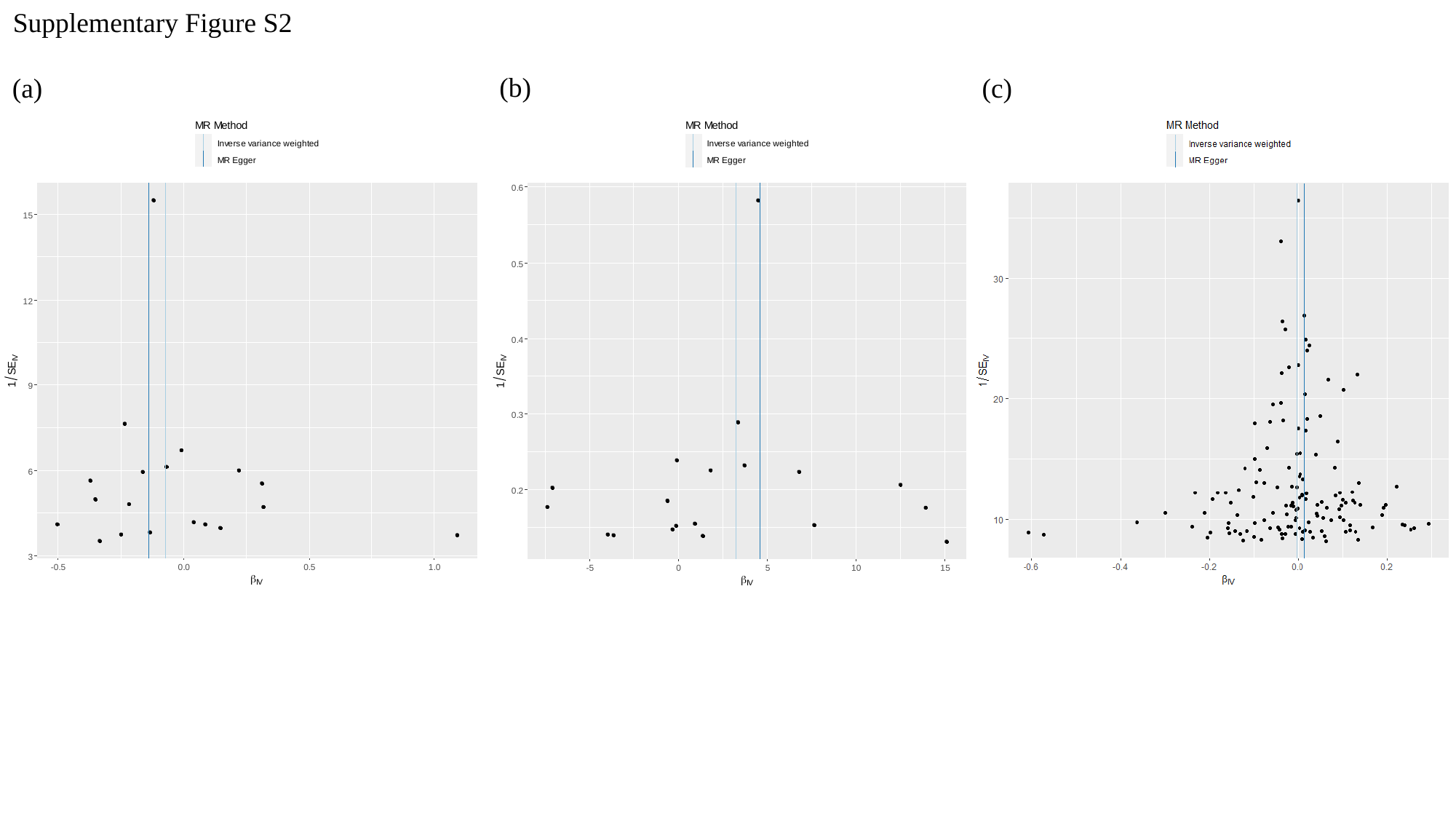

Supplementary Figure S2
(b)
(c)
(a)

Supplement: S2 Fig — (a) Funnel plot for estimating the risk of AF/F on the change in eGFR. (b) Funnel plot for estimating the causal effect of the risk of AF/F on the risk of CKD. (c) Funnel plot for estimating the causal effect of the change in eGFR on the risk of AF/F. Each black point representing an SNP is plotted in relation to the estimate of the exposure on the outcome (x-axis) and the inverse of the standard error (y- axis). Vertical lines show the combined causal estimates using IVW (light blue) and MR- Egger regression (blue) methods. (PPTX) [file pone.0261020.s003.pptx]

## Slide 1
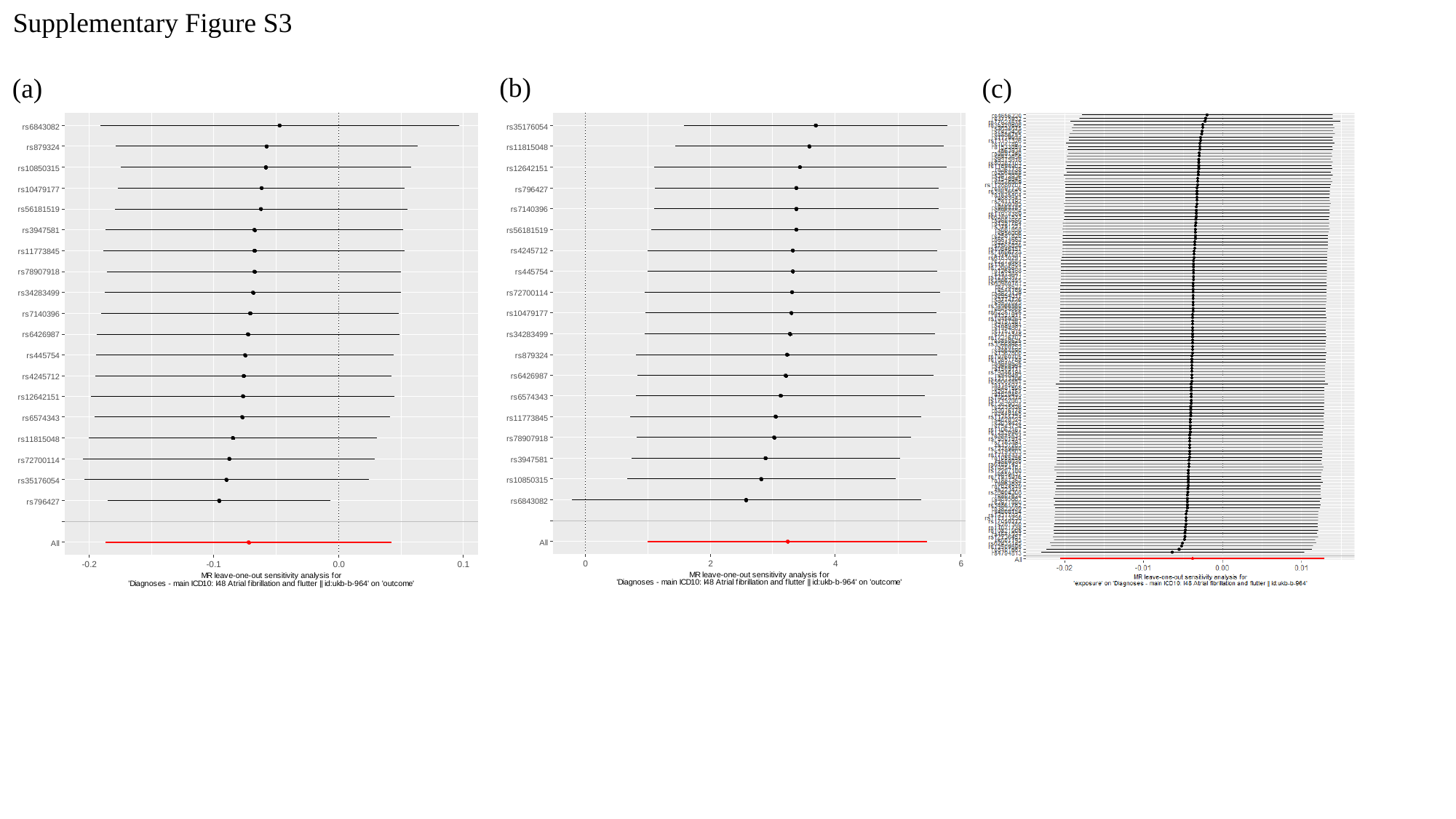

Supplementary Figure S3
(b)
(c)
(a)

Supplement: S3 Fig — (a) Leave-one-out sensitivity analysis for estimating the risk of AF/F on the change in eGFR. (b) Leave-one- out sensitivity analysis for estimating the causal effect of the risk of AF/F on the risk of CKD. (c) Leave-one-out sensitivity analysis for estimating the causal effect of the change in eGFR on the risk of AF/F. Each black point represents the combined causal estimates on the outcome per increase in the exposure using IVW methods with horizontal lines denoting 95% confidence intervals after removing the corresponding SNP from the analysis. (PPTX) [file pone.0261020.s004.pptx]
