# Supplementary material for: Causal effect of atrial fibrillation/flutter on chronic kidney disease: A bidirectional two-sample Mendelian randomization study
Source: PLoS One. 2021 Dec 13;16(12):e0261020. doi: 10.1371/journal.pone.0261020 (PMC8668124; doi:10.1371/journal.pone.0261020)
Supplement: Attachment — Submitted filename: Response to Reviewers.docx [file pone.0261020.s007.docx]

Dear Dr. Jie V Zhao,

We would like to express our sincere gratitude and appreciation for the opportunity to submit a revision of our manuscript entitled "Causal effect of atrial fibrillation/flutter on chronic kidney disease: A bidirectional two-sample Mendelian randomization study".

***Journal Requirements: Please review your reference list to ensure that it is complete and correct. If you have cited papers that have been retracted, please include the rationale for doing so in the manuscript text, or remove these references and replace them with relevant current references. Any changes to the reference list should be mentioned in the rebuttal letter that accompanies your revised manuscript. If you need to cite a retracted article, indicate the article’s retracted status in the References list and also include a citation and full reference for the retraction notice***.

Reference 18 is not "Forthcoming" but "Online ahead of print" and we revised it (please see line 397 in the newest version of the manuscript).

Now we have confirmed that our reference list is correct without retracted papers.

And our responses to each Reviewer’s comment are described below in a point-to-point manner.

We thank you very much for handling this paper and look forward to hearing from you again.

Sincerely,

Masahiro Yoshikawa, M.D., Ph.D.

Corresponding author.

Address; Division of Laboratory Medicine, Department of Pathology and Microbiology, Nihon University School of Medicine, Oyaguchi-kamicho 30-1, Itabashi, Tokyo, Japan.

Phone; 81-3-3972-8111

e-mail: myosh-tky@umin.ac.jp

**To Reviewer #1**

**Our responses to the Reviewer’s comments**

***The authors have adequately addressed my comments*.**

We would like to express our sincere gratitude and appreciation for your insightful suggestions.

We have learned a lot, and we believe that our manuscript has been significantly improved thanks to Reviewer #1.

***Figure 1 is still hard to read. Please revise*.**

Could you please click "Click here to access/download;Figure;Fig1.tif " on the top right of Revised Figure 1 page to download TIF file with a resolution of 600 dpi?

**To Reviewer #2**

**Our responses to the Reviewer’s comments**

***I thank the Authors for their thorough job addressing all my comments. Explanations were provided, corrections were made*.**

We would like to express our sincere gratitude and appreciation for pointing out our mistakes and faults. We also thank you very much for insightful suggestions.

We believe that our manuscript has been significantly improved thanks to Reviewer #2.

***Only one last point I still do not understand and concerns my previous point on palindromic SNPs. While Authors’ explanation are understandable I still have trouble: In Supplementary Table 1, one SNP (rs7853195) is marked as ‘palindromic ambiguous’ and is mentioned to be excluded from the analysis in the Results (line 173). This can be retraced by the fact that the allele frequency of the effect allele is 0.42 in both GWAS of exposure and outcome. However, there is another SNP (rs4245712) in that table that is not marked as ‘palindromic ambiguous’ but has allele frequency of the effect allele of 0.55 (exposure) and 0.56 (outcome). Since MAF is >0.42 (definition of palindromic SNPs, lines 119-120) I do not understand why this SNP was not excluded. In Supplementary Table 2, there are also such instances (e.g., rs1055256). What do I miss? Did the Authors used a different source than the GWAS to define palindromic SNPs or incorporated some additional criteria?***

Thank you very much for your careful reading and suggestion, and we sincerely apologize that our tables were still hard to read.

In the same way as MR-Base platform does, we have marked the palindromic SNPs "TRUE" in the "palindromic" column and the excluded SNPs "TRUE" in the "ambiguous" column in **the newest version of Supplementary Tables 1 and 2**. **Please also see lines 174-175 and 242-243 in the newest manuscript.**

For example, **rs4245712** in **Supplementary Table 1** has effect allele frecency (EAF) of 0.55 for exposure and 0.56 for outcome (i.e. minor allele frequency (MAF) > 0.42), but it is not a palindromic SNP ("palindromic" is "FALSE"). Therefore, it was not excluded.

In **Supplementary Table 2**, EAF of **rs4820324** for exposure is, more precisely, 0.5801 (Please see row 308 and column N in ST4 in the GWAS study by CKDgen, Nat Genet. 2019;51: 957-972, <https://www.ncbi.nlm.nih.gov/pmc/articles/PMC6698888/>). And EAF of **rs4820324** for outcome is 0.580514. Both are slightly > 0.58 (i.e. MAF < 0.42), and therefore it was not excluded from our analyses.

On the other hand, **rs7853195** in **Supplementary Table 1** in our manuscript (EAF is 0.421733 for exposure, slightly > 0.42) was excluded properly.

***Otherwise, I am quite satisfied with the presentation of the project***.

We really appreciate your help in improving our manuscript.
