# Supplementary material for: Causal effect of atrial fibrillation/flutter on chronic kidney disease: A bidirectional two-sample Mendelian randomization study
Source: PLoS One. 2021 Dec 13;16(12):e0261020. doi: 10.1371/journal.pone.0261020 (PMC8668124; doi:10.1371/journal.pone.0261020)
Supplement: Attachment — Submitted filename: Response to Reviewers_PlosOne.docx [file pone.0261020.s008.docx]

Dear Dr. Jie V Zhao,

We would like to express our sincere gratitude and appreciation for the opportunity to submit a revision of our manuscript entitled "Causal effect of atrial fibrillation/flutter on chronic kidney disease: A bidirectional two-sample Mendelian randomization study".

Our response to Reviewer’s comment is described below in a point-to-point manner.

We thank you very much for handling this paper and look forward to hearing from you again.

Sincerely,

Masahiro Yoshikawa, M.D., Ph.D.

Corresponding author.

Address; Division of Laboratory Medicine, Department of Pathology and Microbiology, Nihon University School of Medicine, Oyaguchi-kamicho 30-1, Itabashi, Tokyo, Japan.

Phone; 81-3-3972-8111

e-mail: myosh-tky@umin.ac.jp

**To Reviewer #1**

***Figure 1 is still hard to read. I don't think the author's response that "Could you please click "Click here to access/download;Figure;Fig1.tif " on the top right of Revised Figure 1 page to download TIF file with a resolution of 600 dpi?" is acceptable. If one chooses to print out a hard copy to read, then where to click*?**

We would like to apologize for any inconvenience.

We have separated original Figure 1(a-c) into revised Figures 1-3 to increase the resolution.

We sincerely hope that new Figures could meet with your criteria.
